# Diabetes care and prevention services provided by pharmacists: Progress made during the COVID-19 pandemic and the need for additional efforts in the post-pandemic era

**DOI:** 10.1016/j.rcsop.2022.100137

**Published:** 2022-04-20

**Authors:** Mohamed Hassan Elnaem, Wesley Nuffer

**Affiliations:** a Department of Pharmacy Practice, Faculty of Pharmacy, International Islamic University Malaysia, Kuantan, Pahang, Malaysia; b Quality Use of Medicines Research Group, Faculty of Pharmacy, International Islamic University Malaysia, Kuantan, Pahang, Malaysia; c Department of Clinical Pharmacy, Skaggs School of Pharmacy and Pharmaceutical Sciences, University of Colorado Anschutz Medical Campus, Aurora, CO 80045, USA

**Keywords:** COVID-19, Diabetes, Pharmacists, Control, Prevention

## Abstract

Diabetes is a challenging metabolic disease that significantly impacts people's health worldwide. It requires a comprehensive approach for better prevention and control, especially during challenging times such as the recent pandemic. The COVID-19 pandemic has altered how health care professionals, including pharmacists, provide health care. With the widespread use of virtual and online platforms for service delivery, pharmacist-led diabetes care has been transformed to meet the needs of patients during the pandemic. This article aims to discuss examples of pharmacist-led diabetes care services during the pandemic and highlight areas where additional pharmacist efforts are needed in the post-pandemic era.

## 1 Background

Diabetes is a global disease that affects nearly half a billion individuals, with an alarming projection of cases in several regions to be doubled in a few years.^1^ It is estimated that the worldwide economic impact of diabetes is around 1.3 trillion United States dollars, or approximately 1.8% of global gross domestic product.^2^ Healthy self-care behaviors, which are critical for diabetes prevention and metabolic control, are difficult to maintain under normal circumstances and became even more difficult during the recent pandemic.^3^ Furthermore, patients with diabetes are at an increased risk of contracting viral infections in general, including COVID-19 infections.^4^ Patients with diabetes who contract COVID-19 infection have a significantly higher mortality rate than those who do not have diabetes.^5^ Considering all of these adverse consequences of developing or not controlling diabetes during and after the pandemic highlights the critical role that healthcare providers, including pharmacists, should play in establishing and sustaining diabetes care and prevention services at the community level. With the imposed pandemic restrictions, it was anticipated that virtual care delivery methods would be fully utilized across healthcare systems throughout the world. This article aims to discuss examples of pharmacist-led diabetes care services during the pandemic and highlight areas where additional pharmacist efforts are needed in the post-pandemic era.

### 1.1 The impact of the pandemic on diabetes self-care behaviors

The impact of the pandemic on chronic disease care has been studied, and certain patient groups, such as the unemployed, unmarried, and those with low socioeconomic status, were found to be more vulnerable to pandemic-related stress.^6^ Concerning diabetes care amid the pandemic, stress is thought to worsen individuals' health coping capabilities, directly impacting their ability to achieve proper glycemic control.^7^ Thus, psychological and emotional support for patients at risk of or living with diabetes during the pandemic is critical to avoiding the negative consequences of suboptimal diabetes control.^8^ Moreover, from a disease prevention perspective, health promotion interventions that have been implemented in well-structured diabetes prevention programs have shown great potential to decrease the risk of developing diabetes (50–70%).^9^ Unfortunately, during the pandemic, these health promotion activities were significantly affected by lockdowns implemented globally at different levels.^10^

Considering the seven recommended self-care behaviors for diabetes prevention and management,^11^ the following points were the potential barriers to diabetes care services in the pandemic era:

• Psychological stress and its effect on the healthy coping styles of many individuals
• Changes in diet and exercise habits because of lockdown, movement restriction orders, and extended regulations on gathering activities
• Relative lack of emphasis on disease prevention due to exhausted healthcare resources focused on managing the pandemic and its complications

With these barriers in mind, community pharmacists were well leveraged to play a critical role in diabetes prevention and control by promoting healthy lifestyle behaviors, providing screenings and referral services, and serving as a credible source of public health information.

### 1.2 Pharmacist role in diabetes self-care behaviors

According to the 2020 American Association of Diabetes Care and Education Specialists (ADCES) revised model of diabetes care and education, there are seven self-care behaviors to promote optimal health and quality of life among individuals at risk or living with diabetes.^11^ This model of care has emphasized the role of ongoing evaluation and provides support for individuals in attaining and sustaining the recommended behaviors. In addition, the model design recognized the potential adverse impact of distress from having diabetes on individuals' abilities to adopt the recommended behavior changes and, consequently, the ability to sustain them.^12^ Thus, healthy coping was added as the first recommended behavior change, which has the potential to affect and impact all six other behaviors and the patient's overall quality of life. Health coping refers to an individual's ability to develop and maintain a positive attitude toward disease self-management, including all necessary changes and communication with health care providers.^13^ This behavior change emphasizes the importance of psychological support for diabetes patients as an integral part of diabetes care.^14^ Pharmacists have the potential to play a significant role in supporting, educating, and collaborating with all stakeholders to improve diabetes care outcomes, which is challenging in resource-constrained settings, particularly during the unprecedented pandemic period.^15^^,^^16^ Table 1 demonstrates the pharmacists' roles in supporting patients to adopt the recommended changes in diabetes self-care behaviors.

**Table 1:** Pharmacists' roles in supporting patients to adopt the recommended changes in diabetes self-care behaviors.

Category of Self-care behaviors^11^	Recommended changes^11^	Pharmacists' roles	Examples of interventions
1. Healthy Coping	▪ Increase self-efficacy ▪ Address cognitive impairment ▪ Seek support	▪ Support and interventions to decrease diabetes-related stress and depression	▪ Coordinate peer support^17^ ▪ Mobile phone supported guided web-based intervention^18^
2. Healthy Eating	▪ Personalized meal plan ▪ Healthy eating patterns ▪ Portions measurement ▪ Intake monitoring ▪ Literacy of nutrition fact labels	▪ Individualized interventions for healthy eating literacy that emphasize the importance of good nutrition to health assist patients in recognizing the benefits of adopting healthy eating patterns and help patients develop practical skills for reading food labels and making healthy food choices^19^	▪ Personalized care planning^20^ ▪ Technology-based solutions to simulate daily activities that might help develop the required skills for adopting healthy eating patterns^21^
3. Being Active	▪ Aerobic exercise ▪ Unstructured or daily activity ▪ Decrease sitting time ▪ Address personalized barriers (lack of time, social support and enjoyment, inappropriate starting intensity)	▪ Personalized consultations on addressing barriers ▪ Promoting a culture of regular exercise	▪ Structured interventions of regular exercise (at least 150 min/week) or resistant training or combination^22^
4. Taking Medication	▪ Maintain updated medication list/history ▪ Fill the prescription ▪ As-prescribed medication taking ▪ Share medication-related beliefs and concerns	▪ Provide user-friendly forms for medication list/history ▪ Medication counseling ▪ Engaging with patients to correct false beliefs and address effectiveness and safety concerns ▪ Examine possibilities for simplifying the medication regimen^23^	▪ Using a conceptual model (e.g., the necessity-concerns framework) for better understanding patients' beliefs (essential for tailored interventions)^24^
5. Monitoring	▪ Accurate measures tracking ▪ Records keeping and sharing ▪ Trends identification	▪ Glucose monitoring training ▪ Educate patients on interpreting, following up, and communicating the identified trends to their HC providers	▪ Facilitate study circle among patients with diabetes and be effectively involved in diabetes management programs^25^
6. Reducing Risk	▪ Early action ▪ DSMES participation ▪ Having adequate sleep ▪ Actively engaging in health (sharing data and asking questions)	▪ Actively participating as diabetes educators in DSMES programs ▪ Educate patients on the impact of sleep quality on glycemic control ▪ Provide a private environment and friendly, competent staff to encourage patients' engagement	▪ Structured programs for screening and raising awareness of DM-related complications^16^
7. Problem Solving	▪ Asking clarifications ▪ Disclose challenges ▪ Shared decision making ▪ Collaborative goal setting ▪ Lifelong learning and flexibility in revising plans	▪ Support and follow up on the implemented changes in a collaborative care model ▪ Support in amending plans for better outcomes	▪ Use of shared decision aids^26^ ▪ Proactive, collaborative clinical communications^27^

DSMES = diabetes self-management education and support.

HC = health care.

### 1.3 Patients' preferences for engagement in diabetes self-management education and support (DSMES) offered in community pharmacies

It is imperative for pharmacists to understand the comprehensive nature of diabetes management and the personalized needs of their patients to optimize the care they provide. According to a recent study, while patients were comfortable engaging with community pharmacy-based DSMES programs via various platforms (email, phone calls, and short messages), they preferred to receive written program information, such as brochures and flyers.^28^ More importantly, they preferred to receive support from community pharmacists on healthy eating (i.e., diabetes cooking classes, food selection for diabetics), setting individualized treatment goals, and glucometer training.^28^ In addition, topics concerning healthy behaviors were chosen significantly more frequently than medication-related topics such as medication counseling and insulin injection training. This should help direct and focus community pharmacists' future efforts toward better integrating health promotion support activities into their DSMES programs.

## 2 Progress made during the COVID-19 pandemic

This section discusses several pharmacist diabetes care programs implemented during the COVID-19 pandemic. These examples were chosen to highlight specific characteristics that could be applied in various settings post the pandemic.

### 2.1 Virtual Diabetes/Pre-Diabetes Education Program

As a part of their experiential pharmacy practice experiences and to overcome the social distancing requirements, student pharmacists at the University of Hawai'i at Hilo created a Diabetes Education Webinar (DEW) to provide free, virtual diabetes education classes.^29^ The program was structured as four weekly 30-min classes offered to patients with DM or pre-DM and their family/friends using a virtual platform (Zoom Conferencing). The designed content provided essential information about diabetes, managing the diabetes diet, the importance of activity, and examples of different exercises, medication education, and adherence techniques. The program also offered referral services to develop individual care plans in collaboration with the University Family Medicine Clinic. Furthermore, completion certificates and free pillboxes were also given to people who finished the programs.

### 2.2 Let's Talk about Diabetes program

A program was conducted to utilize the community pharmacy workforce in Saudi Arabia to improve diabetes education.^30^ A total of 25 pharmacists were certified as diabetes educators to provide four 1: 1 counseling sessions, each lasting 20 min, scheduled over a 2-to-4-month period. The program content focused on medications, lab results, and glucose monitoring. The outcomes were positive across 380 participants in improving diabetes awareness attitudes toward diabetes and achieving significant improvement in HbA1c reduction from 8.50 to 7.32% (*P* < 0.001). This one-to-one session model provides an excellent opportunity to consider the personalized nature of the most recommended self-care behaviors.

### 2.3 Pharmacist-Led Group Diabetes Class

Pharmacists within a family medicine clinic in Arkansas conducted once-monthly, 90-min education classes for 66 individuals.^31^ The educational content included dietary and exercise recommendations, the pathophysiology of diabetes, potential complications and monitoring, and common anti-diabetic medications. The intervention was associated with significant improvements in patients' weight (mean decrease = 1.8 kg, *p* = 0.03) and HbA1c lab values (mean decrease = 1.16%, *p* = 0.001). As long as patients were in a group setting for support and sharing, the efficiency and sustainability of this method for giving personalized recommendations for self-care behavior changes may be in question.

### 2.4 Telepharmacy-based interventions

Telepharmacy is defined by the American Society of Health-System Pharmacists (ASHP) as the remote provision of pharmacy services by qualified personnel.^32^ Telepharmacy services have had a well-established care model for more than two decades.^33^ Not unexpectedly, during the pandemic, the overall use of telehealth visits increased significantly, with some US settings reporting a 78-fold increase in utilization.^34^ However, an Indian study found that telehealth was not widely accepted during the pandemic, despite a high level of satisfaction among those who utilized it. This highlights the need for further promotion and facilitation of these services.^35^

It is critical to take advantage of opportunities to maintain and improve access to healthcare in the post-pandemic era, even though the increased use of telepharmacy is expected to decline as the pandemic is brought under control.^36^ During the pandemic, several telepharmacy models of care have been implemented successfully, either as pharmaceutical care associated with medication home delivery services or as pharmacist-led multidisciplinary virtual integrated care clinics.^37^^,^^38^

A recent study on telepharmacy for diabetes care found that most of the interventions were delivered via telephone and were associated with improved access to health care, a reduction in the need for physical consultations, better diabetes control (a significant decrease in HbA1c), and improved medication adherence.^39^ Importantly, those interventions with less patient engagement (a call for 5 min within 2–3 weeks of the intervention) and those compared to other telehealth interventions led by another HC provider, e.g., nurses, were less likely to show a significant impact.^39^ By providing remote value-added services, promoting healthy lifestyle changes, reminding for necessary monitoring and annual screenings, and encouraging patients to maintain continuity of care, telepharmacy enabled pharmacists to demonstrate their empathy-based professional support for diabetes care.^15^ It is critical to emphasize that telepharmacy has the potential to spread rapidly in low- and middle-income countries if proper training, arrangements, and compensation are considered.^40^ Table 2 summarizes the main features of pharmacy-led programs in the pandemic that could be beneficial in sustaining diabetes care in the post-pandemic era.

**Table 2:** Main features of effective pharmacist-led diabetes care programs.

Feature	Description and considerations
Delivery platform	▪ Face-to-Face, Zoom conferencing, telephone calls, messages, and emails ▪ The choice should be based on patients' preferences
Time and engagement	▪ At least 20 min up to 90 min ▪ Less than 20 min might be associated with less opportunity for proper engagement
Content	▪ Tailored according to patients' needs ▪ Typically includes comprehensive disease education, healthy behaviors, medication counseling, and self-monitoring training
Group or individual	▪ Individual-based is more suitable for personalized recommendations ▪ Group-based provides better support and sharing opportunities ▪ Hybrid: start by group-based, then arranging individual sessions might be an appealing option to meet different requirements
Collaborative (design)	▪ Collaborative care in the intervention design is most likely associated with improved outcomes
Collaborative (referral)	▪ Collaborative care by coordinating and providing referral services
Participants' incentives	▪ Participants should be maintained motivated and engaged ▪ Incentives or simple gifts may be considered accordingly

## 3 The need for additional efforts in the post-pandemic era

### 3.1 Screening, detection, and referral services

According to the latest statistics of the International Diabetes Federation, there are approximately 230 million individuals with undiagnosed diabetes. The highest proportion of these cases are in Africa (60%), followed by Southeast Asia (57%), Western Pacific (56%), MENA region (45%), Europe (41%), and finally, North America and the Caribbean (38%).^1^ Despite the wide availability of risk assessment tools, there is still a global estimated average of 50% of diabetes patients who are yet undiagnosed, which is an alarming statistic.41., 42, 43. There is a need for better utilization of pharmacists, as the most accessible healthcare providers, to play a more significant role in providing better screenings, detection, and referral services to these patients. The screening services should encompass risk factors (such as family history, obesity, and physical inactivity), signs (such as acute metabolic deterioration and severe dehydration), and symptoms (such as polyuria, polydipsia, and polyphagia), in addition to point-of-care glucose and HbA1c levels.^44^

One meta-analysis evaluated the effectiveness of diabetes screening services and found that pharmacies are viable sites for effective screening and detection of diabetes. However, the sustainability of such services was frequently challenged by the extent of referral uptake, making it necessary to establish strategies for increasing uptake to follow-up testing.^45^ One critical observation in the meta-analysis was that the vast majority (94%) of the studies included were conducted primarily in developed countries (the United Kingdom, the United States of America, Canada, and Australia), which have a lower prevalence of undiagnosed diabetes compared to developing countries in Africa and Southeast Asia.^45^ This highlights pharmacists' disproportional efforts in screening services, which are lacking in countries with the highest percentage of undiagnosed diabetes, and emphasizes the need for well-structured, widely available screening services by pharmacists, particularly in these countries.^1^ Varying defined scopes of practice for pharmacies and reimbursement opportunities for services across different countries may limit their abilities to engage in this work. Moreover, successful implementation of screening services in community pharmacies should carefully consider the pharmacist staff engagement, pharmacy staffing, operationalization, and external engagement with stakeholders, i.e., consumers and doctors.^46^ Thus, community pharmacies have various needs and opportunities to serve as centers for diabetes prevention and screening, provided that well-structured service models are designed and implemented to engage all relevant stakeholders and pharmacists are compensated for their time spent on this critical work.

### 3.2 Pharmacies as centers for credible public health information

Tremendous scientific contributions were conducted, posted, and shared during the pandemic unprecedentedly. So much information coming out in such a short period presented challenges for scientific journals, reviewers, and the medical community to scrutinize and evaluate the information properly.^47^ Patients sought information regarding the evolving pandemic, including questions about mask-wearing and vaccination safety and efficacy. Concerning diabetes management, patients are always encouraged to regularly meet with their health care providers, including pharmacists, to consult, disclose challenges and share their concerns regarding their health.^11^ Due to their overall accessibility, from a public health perspective, pharmacists are optimal as a source of patient education and dissemination of credible information in the community.^15^ Although this educational public health role is logically expected from community pharmacists, we must acknowledge that the retail nature of some practice models can significantly impact the quality of patient-centered educational interventions.^48^ A Canadian study has highlighted the wide gap between the ideal and actual pharmacists' involvement in health promotion activities due to several organizational barriers such as lack of time, staffing, resources, and financial reimbursement.^49^ This demonstrates that even pharmacists' most anticipated professional roles cannot be fully utilized unless appropriate facilitators of this practice are carefully considered. To prepare pharmacists for this public health role, training must be provided to boost pharmacists' confidence in developing their pharmacies as information hubs for public health.^50^ Along with proper training, establishing a service delivery model and promoting service to the general public will assist in broadening the scope of this critical service.^51^

## 4 Conclusion

Pharmacists effectively contributed to the overall optimization of diabetes care and prevention during the pandemic through face-to-face, telehealth/telepharmacy, and virtual platforms to spread awareness & support for diabetes prevention and control and promote healthy behavior changes. However, the role of pharmacists in diabetes screenings, referral services, and providing credible public health information can be further improved by considering the facilitators and determinants of successful implementation of these services at a wider scope.

Conflict of interest statement: authors declare that no conflict of interests was associated with undertaking this work.

## Funding information

There was no specific fund received to conduct this work.
